# Coronaviruses in wild birds – A potential and suitable vector for global distribution

**DOI:** 10.1002/vms3.360

**Published:** 2020-09-24

**Authors:** Md. Mijanur Rahman, Asma Talukder, Mohammed Mehadi Hassan Chowdhury, Reshma Talukder, Rekha Akter

**Affiliations:** ^1^ Department of Microbiology Noakhali Science and Technology University Noakhali Bangladesh; ^2^ Department of Biotechnology and Genetic Engineering Noakhali Science and Technology University Noakhali Bangladesh; ^3^ Department of Architecture State University of Bangladesh Dhaka Bangladesh; ^4^ Department of Biochemistry and Molecular Biology University of Chittagong Chattogram Bangladesh

**Keywords:** coronaviruses, deltacoronavirus, gammacoronavirus, global distribution, wild birds

## Abstract

The recurrent appearance of novel coronaviruses (CoVs) and the mortality and morbidity caused by their outbreaks aroused a widespread response among the global science community. Wild birds' high biodiversity, perching and migratory activity, ability to travel long distances and possession of a special adaptive immune system may make them alarming sources of zoonotic CoV‐spreading vectors. This review gathers the available evidence on the global spread of CoVs in wild birds to date. The major wild birds associated with different types of CoVs are Anseriformes, Charadriiformes, Columbiformes, Pelecaniformes, Galliformes, Passeriformes, Psittaciformes, Accipitriformes, Ciconiiformes, Gruiformes and so on. However, the main type of CoVs found in wild birds is gammacoronavirus, followed by deltacoronavirus. Consequently, it is imperative to enable thorough research and continuous monitoring to fill the study gap in terms of understanding their role as zoonotic vectors and the frequent appearance of novel CoVs.

## Graphical abstract

Globally, coronaviruses (CoVs) are commonly distributed in wild birds, particularly in Anseriformes, Charadriiformes, Columbiformes, Pelecaniformes, Galliformes, Passeriformes, Psittaciformes, Accipitriformes, Ciconiiformes, Gruiformes, and so on. The main type of CoV found in wild birds is gammacoronavirus, followed by deltacoronavirus. The presence of infectious bronchitis virus‐like and avian CoVs has also been recorded. Wild birds may play an important role in the emergence and spread of novel CoVs.

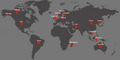

## 1 INTRODUCTION

Coronaviruses (CoVs) are worldwide in distribution, infectious for a wide assortment of warm‐blooded animals and birds, profoundly irresistible, and hard to control given their vast genetic variation, short generation time, and high mutation rates, which can inevitably prompt the rise of new viruses. Such new pathogens can have new highlights that even empower them to change hosts (Woo et al., 2009). Apart from these, because of expanding human–animal interface interactivity, novel CoVs are probably going to rise intermittently in people secondary to cross‐species contaminations and incidental overflow occasions. They are zoonotic in genesis, and the course of infection varies massively from asymptomatic to serious sickness in the respiratory, enteric, hepatic and neurological systems (Cui et al., 2019). CoVs were not viewed as highly pathogenic to people until the appearance of severe acute respiratory syndrome coronavirus (SARS‐CoV) and Middle East respiratory syndrome coronavirus (MERS‐CoV) (Bonilla‐Aldana et al., 2020). CoVs were included in the Blueprint list of priority diseases in 2018 by the World Health Organization (WHO); given their capability to cause public health crises of worldwide concern, and the nonappearance of strong medications and vaccines, these maladies are considered to require expedited innovative research and development (Cui et al., 2019; WHO, 2020a, 2020b; Zhu et al., 2020). CoV has become a global concern again in the current pandemic of COVID‐19 brought about by SARS‐CoV‐2, a novel CoV first identified in December 2019, when a group of patients with pneumonia of obscure origin was connected to a wholesale seafood market in Wuhan, China (Zhu et al., 2020). According to the “Coronavirus disease 2019 (COVID‐19) Situation Report‐101” by WHO, published on 30 April 2020, SARS‐CoV‐2 has taken 217,769 (7.05%) lives out of 3,090,445 confirmed cases, and the death toll is still rising.

Birds are natural pools for supplying viral genes during the development of new species and viruses for interspecies transmission. These warm‐blooded vertebrates show high species biodiversity, perching and migratory conduct, ability to fly long distances and possession of a remarkably versatile immune system, which are ideal qualities for asymptomatic shedding, dispersal and blending of various viruses for the development of novel mutant, recombinant or reassortant RNA viruses. The expanded incursion of people into wildlife habitats and congestion of various natural species in wet markets and ranches have likewise encouraged interspecies transmission among various animals (Chan et al., 2013). Throughout the years wild birds have been under epidemiological observation because these act as a natural repository of many growing zoonotic pathogens and, therefore, significantly affect public health (Miłek & Blicharz‐Domańska, 2018). They are pervasive and exceptionally versatile potential hosts equipped for moving viruses past topographical and political boundaries and have been implicated in the spread of profoundly pathogenic H5Nx avian influenza viruses, tick‐borne encephalitis virus, West Nile virus, Newcastle disease virus (NDV) and influenza A virus (IAV) (Hepojoki et al., 2017).

Regular interspecies spillover of CoVs occurs to new hosts, with SARS‐CoV and MERS‐CoV being the most noteworthy examples of spillover into humans. Bovine CoV, canine respiratory CoV, dromedary camel CoV and even human CoV OC43 all probably come from the same common ancestor, demonstrating substantial host versatility (Lu et al., 2017; Nathalie et al., 2016; Vijgen et al., 2005). SARS‐CoV possibly originated in bats, whereas Porcine Diarrhea CoV may, interestingly, have emerged in birds (Lau et al., 2005; Ma et al., 2015). The 2003 SARS‐CoV outbreak deeply influenced the global medical, economic and social spheres, revitalizing interest in CoV research. One crucial discovery was the recognition of bats as the natural repository for viruses and of civet and other mammals as SARS‐CoV intermediate amplifying hosts. The subsequent advent of MERS‐CoV marked a new era in CoV research history (Lau et al., 2005). Such findings reshaped the "hunting" approach for novel CoVs and reshaped the classification of CoVs on the basis of theirs updated phylogeny and the crucial function of bats in the inter‐ and intra‐species transmission of CoVs (Chan et al., 2013).

In this review, we present an updated scenario on the distribution of CoVs in wild birds worldwide to emphasize their role as a natural pool and possible potential zoonotic vector in spreading and evolving novel CoVs.

## 2 CORONAVIRUSES

CoVs consist of a family of the order Nidovirales (Coronaviridae family). The CoV genome is among the largest genomes of viral RNA (25–32 kb) (Flint et al., 2015). CoVs are classified into four separate genera, based on phylogenetic analysis: alpha‐, beta‐, gamma‐, and delta‐CoV. Mammals bear the alpha‐ and beta‐CoVs, whereas the gamma‐ and delta‐CoVs primarily infect birds, with few deviations. This can be further subclassified into lineages A, B, C and D within beta‐CoV (De‐Groot et al., 2012; King et al., 2011). In 2018, these four lineages were reclassified as beta‐CoV subgenera and renamed embecovirus (former lineage A), sarbecovirus (former lineage B), merbecovirus (former lineage C) and nobecovirus (former lineage D). This also contained a fifth subgenus, hibecovirus (ICTV 2019; Wong et al., 2019). The major factors contributing to the high genetic diversity of CoVs are the large genomes, the RNA‐dependent polymerase infidelity, and the high frequency of homologous RNA recombination (Denison et al., 2011; Jackwood et al., 2012; Woo et al., 2009).

## 3 GLOBAL DISTRIBUTION OF CORONAVIRUSES

**FIGURE 1:**
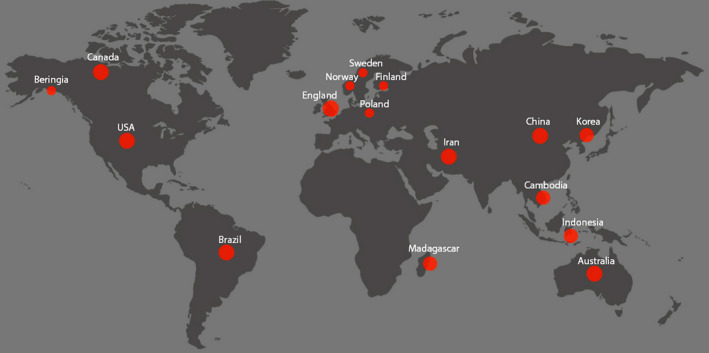
Wild birds across the world from different ecology, climate and geography are carrying coronaviruses (based on the Google world map)

**FIGURE 2:**
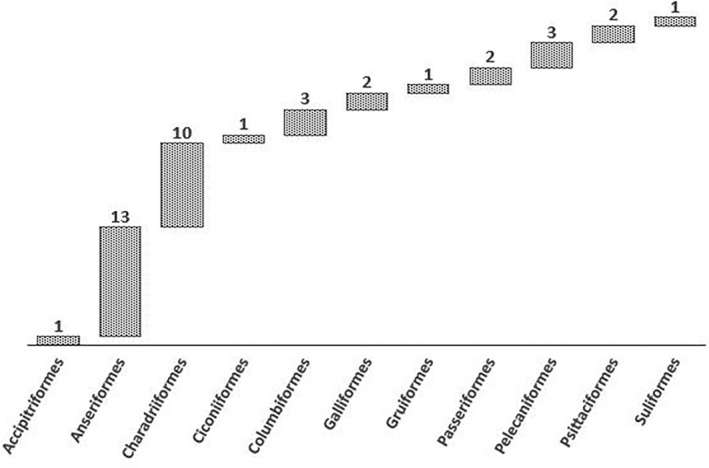
Distribution of major wild birds (order) detected with coronaviruses in studied countries worldwide

Wild birds from all continents except Antarctica are known to carry CoVs (Figure 1). The major wild birds found with different types of CoVs in the studied countries are Anseriformes (12, 75%), Charadriiformes (9, 56.2%), Columbiformes (3, 18.7%), Pelecaniformes (3, 18.7%), Galliformes (2, 12.5%), Passeriformes (2, 12.5%), Psittaciformes (2, 12.5%), Accipitriformes (1, 6.25%), Ciconiiformes (1, 6.25%), Gruiformes (1, 6.25%) and Suliformes (1, 6.25%) (Figure 2). Most of the wild birds from reported countries were found to be positive with the presence of gamma‐CoV (10, 62.5%), followed by delta‐CoV (6, 37.5%), apart from these, infectious bronchitis virus (IBV)‐like CoV (4, 25%), avian CoV (AvCov) (1, 6.25%), gull coronavirus (GuCoV) B29 (1, 6.25%) and IBV (Mass, Conn) (1, 6.25%) also documented (Table 1).

**TABLE 1:** A brief overview of the studies conducted on the prevalence and distribution of Coronaviruses in wild birds all over the world

Countries	Birds (Order)	Coronavirus detected (%)	Name of coronaviruses	References
Hong Kong	Passeriformes	1.1	Deltacoronaviruses	Woo et al. (2012)
	Unknown			
	Anseriformes	50.89	Gammacoronaviruses	Chu et al. (2011)
		4.46	Deltacoronaviruses	
	Pelecaniformes	36.36	Deltacoronaviruses	
	Suliformes	54.17	Deltacoronaviruses	
	Passeriformes	1.8	Deltacoronaviruses	Woo et al. (2009)
Cambodia	Anseriformes	3.03	Gammacoronaviruses	Chu et al. (2011)
	Pelecaniformes	13	Deltacoronaviruses	
Korea	Anseriformes	0.95	Gammacoronaviruses	Kim and Oem (2014)
Iran	Galliformes	8.99	Gammacoronaviruses	Yaghoubi et al. (2019)
Indonesia	Psittaciformes	—	IBV‐like Coronaviruses	Suryaman et al. (2019)
England	Anseriformes	1.6	IBV‐like Coronaviruses	Hughes et al. (2009)
	Charadriiformes			
Finland	Anseriformes	15.56	Gammacoronaviruses	Hepojoki et al. (2017)
	Charadriiformes	9.62	Gammacoronaviruses	
		5.56	Deltacoronaviruses	
	Columbiformes	3.57	Gammacoronaviruses	
Poland	Anseriformes	3.5	IBV‐like Coronaviruses	Domanska‐Blicharz et al. (2014)
	Charadriiformes	2.3		
	Galliformes	17.6		
Norway	Anseriformes	24.4	Avian Coronaviruses	Jonassen et al. (2005)
	Columbiformes	2		
Sweden	Anseriformes	_	IBV‐like Coronaviruses	Muradrasoli et al. (2009)
	Anseriformes	12	Gammacoronaviruses	Wille et al. (2015)
	Anseriformes	18.7	Gammacoronaviruses	Wille et al. (2016)
	Charadriiformes			
	Anseriformes	0.3	Gammacoronaviruses	Wille et al. (2017)
USA	Anseriformes	12.35	Gammacoronaviruses	Paim et al. (2019)
		2.2	Deltacoronaviruses	
	Accipitriformes	0.17	Deltacoronaviruses	
	Charadriiformes	_	Gammacoronaviruses	Jordan et al. (2015)
Canada	Charadriiformes	10	novel GuCoV B29	Canuti et al. (2019)
Brazil	Anseriformes	0.8	Gammacoronaviruses	Barbosa et al. (2019)
	Charadriiformes		Deltacoronaviruses	
	Psittaciformes	_	Deltacoronaviruses	Duraes‐Carvalho et al. (2015)
	Columbiformes	50	IBV (Mass, Conn)	Felippe et al. (2010)
Australia	Anseriformes	15.3	Gammacoronaviruses	Chamings et al. (2018)
	Charadriiformes		Deltacoronaviruses	
Madagascar	Gruiformes	7.8	Gammacoronaviruses	Lima et al. (2015)
	Passeriformes			
	Ciconiiformes			
	Anseriformes			
	Charadriiformes			
Beringia	Anseriformes	6.4	Gammacoronaviruses	Muradrasoli et al. (2010)
	Charadriiformes			
	Pelecaniformes			

### 3.1 China and Hong Kong

A study conducted by Chu et al. (2011) in China showed that 12% of screened, apparently healthy, wild aquatic birds (Anseriformes, Pelecaniformes and Ciconiiformes) were found with gamma‐CoV and delta‐CoV. Gamma‐CoVs were present mainly in birds of Anseriformes, whereas delta‐CoV was identified in birds of Ciconiiformes, Pelecaniformes and Anseriformes in this analysis. The authors found that gamma‐CoV interspecies transmissions between duck species were frequent. In contrast, delta‐CoV may have host specificities that are more stringent. The avian viral and host sequences of mitochondrial DNA also suggest that some CoV may have coevolved with birds of the same order, but not all. A total of 658 samples were tested and gathered in Hong Kong. Ninety‐nine (15%) of those samples were positive for CoV reverse transcription (RT)‐PCR. Both CoVs found in this study were listed as gamma‐CoV and delta‐CoV in phylogenetic terms (Chu et al., 2011). Two independent reports published in 2009 and 2012 by Woo et al. indicated the existence of delta‐CoV (1.8% and 1.1%, respectively) in Hong Kong's Passeriformes birds.

In recent years, In recent years, the understanding of viral diversity has been increased significantly due to the rapid discovery of novel viruses utilizing next‐generation sequencing technologies particularity DNA‐Seq and RNA‐Seq (Chen et al., 2013). Using RNA‐Seq, Chen et al. (2013) described a novel duck CoV within the gamma‐CoV family, distinct from chicken IBV, as shown by sequences in the viral 1b gene from three regions. A survey of domestic Chinese fowls using RT‐PCR targeted to the viral nucleocapsid gene found a total of 102 positive CoVs. Besides, the findings presented novel data supporting the notion that certain host‐specific CoVs other than IBVs circulate in ducks, geese and pigeons and suggested that the novel duck‐specific CoV found in this study by RNA‐Seq is genetically closer to certain CoVs that circulate in wild waterfowl (Chen et al., 2013).

### 3.2 Cambodia

Meanwhile, in Cambodia, Chu et al. (2011) collected cloacal swabs (263) from pond herons, lesser whistling ducks and ruddy‐breasted crakes. CoV‐positive reactions were observed in pond herons at 13.0% (16/123) and lesser whistling ducks at 3.0% (1/33).

### 3.3 Korea

Kim and Oem (2014) analysed the oropharyngeal swabs of 32 species of wild birds. The 14 avian CoVs found belonged to the gamma‐CoV and shared homology with some previously described strains in wild waterfowl but not with IBVs, showing a high‐nucleotide sequence identity. Of the 1,473 samples analysed, 14 (0.95%) were positive. The authors found CoVs in two species of waterfowl: 1 of 96 northern pintails (*Anas acuta*; 1%) and 13 of 361 Indian spot‐billed ducks (*Anas poecilorhyncha*; 3.6%). The partial viral RdRp sequences were determined from 14 CoVs and compared with those from 32 other CoVs of interest. All detected CoVs were phylogenetically categorized as gamma‐CoV along with 2 Korean IBV strains (SNU8067 and KM91), and the 14 Korean CoVs’ RdRp segment showed more than 93% homology sequence (Kim & Oem, 2014).

### 3.4 Iran

In 2015, for the first time in Iran, Yaghoubi et al. (2019) conducted a study to detect gamma‐CoVs in the bird parks in Tehran. The detection rate in bird species such as chicken (15%), pheasant (8.8%), turkey (27.3%), partridge (4.2%) and quail (7.7%) had specific prevalence levels of approximately 8.99% where gamma‐CoVs were identified.

### 3.5 Indonesia

In Bogor, West Java, Indonesia, an IB‐like avian CoV was isolated from healthy *Eclectus* parrots (*Eclectus roratus*) belonging to a bird breeder (Suryaman et al., 2019). The parrot *Eclectus* is a native bird to Indonesia and Northern Australia and is mostly kept as a pet. The similarity between captive bird‐isolated viruses and those in domesticated poultry was troubling because there was an indication that a reverse spillover effect had already occurred from poultry farms into the ecosystem. Wild birds and other non‐Galliform Aves may harbour IBV or IB‐like CoV without displaying any symptoms and reflect the wide variety of CoV in the host.

### 3.6 England

Wildfowl (Anseriformes) and waders (Charadriiformes) were recorded in England for carrying CoVs (Hughes et al., 2009). CoV RNA was observed in 7 faecal sample pools, providing an approximate prevalence of 1.6% at individual bird level. Of those pools with positive CoV outcomes, four were obtained from ducks. Another pool had samples of whooper swans (*Cygnus cygnus*), one sample of red knots (*Calidris canutus*) and one sample of Eurasian oyster catchers (*Haematopus ostralegus*). PCR‐positive pools were in the estuarine, salt marsh or standing water environments from birds sampled. All the birds were relatively well. Although samples from wild bird populations comprising 46 species from various and diverse habitats were collected, CoV RNA was only identified in wildfowl (Anseriformes) and waders (Charadriiformes) (Hughes et al., 2009).

### 3.7 Finland

Hepojoki et al. screened 939 samples of 61 bird species over 4 years (2010–2013) using a standard, conserved RT‐PCR targeting a 179 fragment of the RdRp gene (Orf1ab) of all wild bird lineages in Finland where prevalence was up to 11%, which is relatively high. A total of 5.4% (51/939) of birds tested were found positive for CoV RNA, of which 27 were found to be healthy and 24 were found to be dead or ill. In 8 species (*Anas crecca*, *Anas platyrhynchos*, *Cygnus cygnus*, *Clangula hyemalis*, *Chroicocephalus ridibundus*, *Larus argentatus*, *Larus fuscus*, and *Columpa* sp.), CoV RNA was found. CoV RNA was most abundant in the samples from 2010 (11%) and 2013 (7.2%), although only a few of the samples tested were found positive for 2011 (0%) and 2012 (0.7%). Four of the CoV‐positive samples were even detected with IAV (Hepojoki et al., 2017).

### 3.8 Poland

In 2014, Domanska‐Blicharz et al. was the first to identify various fragmentary IBV‐like genes in wild bird populations. Between 2008 and 2011, they studied 884 wild birds primarily from the Anseriformes, Charadriiformes and Galliformes orders for CoV‐like IBV in Poland. CoV was found in 31 (3.5%) of the birds studied, with detection rates in Anseriformes of 3.5%, Charadriiformes of 2.3% and Galliformes of 17.6%. Just 10 of the 31 positive samples yielded positive results in molecular experiments targeting specific IBV genome fragments: 5 samples were positive for the RdRp gene, 4 for gene 3, 8 for gene N and 8 for the 3′‐untranslated region fragment. The majority of fragment genes identified tended to be IBV‐like genes of the most frequently identified IBV lineages in the geographic area (i.e. Massachusetts, 793B and QX). Two waves of CoV infections were detected – one in spring (April and May) and the other in late autumn (October to December) (Domanska‐Blicharz et al., 2014).

### 3.9 Norway

A Norwegian study by Jonassen et al. (2005) recorded that the prevalence of CoV RNA among graylag goose (*Anser anser*) in Northern Europe was as high as 38% in 2004, but the authors also noted broad annual and geographical variations; in 2003, the prevalence was only 18%. Screened were the bird populations of *Anser anser*, wild pigeon (*Columbia livia*) and mallard (*Anas platyrhynchos*). In sampled birds, 40 of 163 were CoV‐positive in the graylag goose, whereas 2 of 100 sampled pigeons and 1 of 5 sampled mallards tested positive. The infected graylag geese showed reduced body weights compared with virus‐negative species, suggesting the clinical importance of the infection (Jonassen et al., 2005).

### 3.10 Sweden

CoV infections in mallard ducks (*Anas platyrhynchos*) were also recorded in Sweden, with one of the studies showing a prevalence of CoV infections with the seasonal variation of 6.9%. The prevalence of CoVs among wild waterbirds in Sweden was reported by Wille et al. (2016) to be only 18.7%, which is higher than many other wild bird surveys. In this study, a total of 764 birds from 11 Anseriform species (ducks, geese, swans) and 11 species of Charadriiformes (gulls, terns, shorebirds) were sampled. However, the organisms, groups and orders were poorly described. Diving ducks were found with the highest prevalence (39%). Although the sample size was small (37), greater scaup (*Aythya marila*) had a prevalence of 51.5%. Additionally, dabbling ducks of the genus *Anas were* found with high prevalence. Particularly, mallard had a prevalence of 19.2%. Whereas Anseniformes was found with the highest prevalence, black‐headed gull (*Chroicocephalus ridibundus*) but not in the intern and wader species from the order Charadriiformes was detected with CoV. Gamma‐CoVs were detected in 11 mallards and 3 scaups have been sequenced (Wille et al., 2016). The other two studies by Wille et al. (2017) and Wille et al. (2015) revealed 12% and 0.3%, respectively, for gamma‐CoV in Anseriformes birds. Muradrasoli et al. (2009) also recorded IBV‐like CoV in Anseriformes birds.

### 3.11 USA

Jordan et al. (2015) analysed 133 pooled samples of wild aquatic birds in the United States, wherein only one of ruddy turnstone (*Arenaria interpres*) found positive with CoV, showing nucleotide sequence similarity to duck CoV (DK/CH/HN/ZZ2004) and suggesting a likely low prevalence of CoVs in wild aquatic birds in the eastern half of the United States. Nonetheless, Paim et al. (2019) sought to determine the occurrence of delta‐ and gamma‐CoVs in wild terrestrial and aquatic migratory birds in Arkansas, Illinois, Indiana, Maryland, Mississippi, Missouri, Ohio, Tennessee and Wisconsin in a comparative way. A total of 1,236 cloacal/faecal swabs were collected over the 2015–2018 period. With up to 29 positive samples per state, a total of 61 (4.99%) samples were CoV positive. Unlike previous Asian studies, gamma‐CoVs were seen to be more prevalent in the United States than delta‐CoVs, indicating that the latter can spread in birds with lower performance. This may imply the evolving status of delta‐CoV and its incomplete adaptation to new host species, restricting their spread (Paim et al., 2019).

### 3.12 Canada

Canuti et al. (2019) reported that a novel GuCoV (B29) was identified in great black‐backed gulls (*L. marinus*) (3/26, 11.5%) and American herring gulls (*Larus argentatus smithsonianus*) (2/24, 8.3%) belonging to the Charadriiformes order. GuCoV B29’s phylogenetic analysis indicated that this virus may represent a novel species within the gamma‐CoV family, similar to other novel CoV species described recently (Canuti et al., 2019).

### 3.13 Brazil

Despite Brazil having 18% of the diversity of global avian species, research on the prevalence of avian viral diseases in wild birds in South America is scarce. Barbosa et al. (2019) carried out a retrospective study of the existence of CoVs in 746 wild birds whereby CoV RNA was observed and sequenced from six samples (0.8%), three of which were linked to gamma‐CoV and the other three to delta‐CoV. The research evidence indicates the finding of avian gamma‐ and delta‐CoV in birds obtained in the south‐eastern and southern regions of Brazil, more precisely in Sao Paulo and the state of Rio Grande do Sul. Sampled in Ibirapuera Park, downtown Sao Paulo, southeast Brazil, CoV RNA was found in three Chinese geese (*Anser cygnoides*). One sample revealed similarity (93%) with sequences from gamma‐CoV viruses that are related only to migratory birds, whereas the other two (84%) belonged to delta‐CoV. Despite these sequences being closely similar to those of wild bird CoVs, this category also contains ferret, pig and leopard CoVs, showing the capacity for spillover events between bird and mammal hosts of CoVs (Barbosa et al., 2019). Other studies also found delta‐CoV from Psittaciformes (Duraes‐Carvalho et al., 2015) and IBV (Mass, Conn) from Columbiformes birds in Brazil (Felippe et al., 2010).

### 3.14 Beringia

A study by Muradrasoli et al. (2010) carried out in the Beringia (comprising areas of Alaska and Siberia) indicated gamma‐CoVs RNA in 6.4% of the birds tested (Anseriformes, Pelecaniformes and Charadriiformes). Some of the sequences found in the Chinese sample were intriguingly similar to those found in the Beringia region. Samples from 26 bird species were tested with RT‐PCR for the CoVs polymerase (RdRp) gene, and 64 of the 1,002 faecal and cloacal samples were positive (6.4%). Positives of 18 species were identified. The researchers divided the species into six groups, which represented both their taxonomy and ecology. Such groups were geese (5 species), waders (9 species), gulls (6 species), ducks (1 species), auks (2 species) and seabirds (3 species). All 18 CoV‐positive bird species contained gamma‐CoV, and there was a major variation in PCR positivity among bird groups. Wader species were more often recorded as CoV positive (17.1%), followed by ducks (11.5%), geese (8.2%), gulls (3.1%) and seabirds (1.5%), although auks had the lowest prevalence (0.8%). By comparison, none of the 101 tufted puffins studied were CoV positive. CoVs in Beringia are common among wild birds, and their regional distribution and frequency are higher than previously thought.

### 3.15 Australia

Chamings et al.’s (2018) study of CoVs, the first‐ever report on Australian wild birds, in which different sampling locations were used, revealed the presence of 15.3% (141/918) CoVs in samples from duck species, shorebirds and herons. Sequencing of chosen positive samples found mostly gamma‐CoV but some delta‐CoV as well. Australian duck gamma‐CoVs were similar to duck gamma‐CoVs around the world based on the highly conserved Orf1 PCR amplicon sequencing. Some sequenced gamma‐CoV shorebirds belonged to lineages of Charadriiformes, whereas some were more closely related to gamma‐CoV pigeons. Australian duck and heron delta‐CoVs belonged to lineages of other duck and heron delta‐CoVs, which were available in the nucleotide sequence and were approximately 20% distinct from other delta‐CoV sequences. Sequences of shorebird delta‐CoVs formed a lineage with a delta‐CoV from a ruddy turnstone found in the United States. This indicates that Australian ducks’ gamma‐CoVs are extremely similar to those seen in other areas, and because Australian ducks seldom come into contact with migratory Palearctic duck species, it has been speculated that migratory shorebirds are the main vector for bringing wild bird CoVs to and from Australia (Chamings et al., 2018).

### 3.16 Madagascar

Lima et al.’s (2015) analysis confirmed the existence of CoVs in Madagascar's wild birds based on targeting a conserved sequence of genomes among various groups of CoVs. The existence of gamma‐CoVs in different species of Anseriformes, Charadriiformes, Ciconiiformes, Gruiformes, and Passeriformes was revealed by phylogenetic analyses. Furthermore, several sequences linked to specific strains of IBV. To determine CoVs plolymerase RdRp gene, samples from 17 bird species were investigated, and 28 of the 357 cloacal samples were figured out as positive (7.8%). Positives were found in 11 different species. The findings suggest that genetically divergent avian CoVs circulate among numerous wild bird species at Alaotra Lake, Madagascar (Lima et al., 2015). This lake has a rich wildlife ecosystem with a fairly large number of endemic, uncommon and endangered species (Ferry et al., 2009; Guerrini et al., 2014). Given the results in the samples examined here on the identification of avian CoVs, it may be concluded that these viruses are common among birds found in this area. The identification of gamma‐CoV sequences in this area of study, and geographically distinct regions such as Russia, Alaska and Cambodia, do indeed suggest that CoVs are common among birds associated with water ecosystems, raising concerns about potential consequences for wildlife and poultry development (Lima et al., 2015).

## 4 ROLE OF MIGRATORY BIRDS IN THE DISSEMINATION OF CORONAVIRUSES

Migratory birds may play a significant role in the survival and dissemination of CoV in nature, illustrating their possible contribution to the advent of new CoV diseases in wild and domestic birds (Lima et al., 2015). Hepojoki et al. (2017) identified that Finnish duck CoVs are highly closely related to gamma‐CoVs identified in ducks from Siberia and China that are connected by migratory routes. The close genetic association between these strains (also those reported in South Korea and England) suggests that migratory birds have a particular role to play in dispersing CoV to different geographic locations. Nevertheless, the study of the sequence may be somewhat skewed due to the limited supply of reference strains. Researchers have concentrated mainly on Northern hemisphere duck and shorebird populations, but the presence of other avian species and a broader global range of avian CoVs remain uncertain (Hepojoki et al., 2017). Remarkably, phylogenetic similarities suggest coastal migration from Africa to Madagascar among most of the sequences found in Madagascar and others in Asia. The main path of waterbird migration to Madagascar is from the Eastern African wetlands (Dodman & Diagana, 2006). Three samples were collected from migratory birds caught in watering areas at the Lagoa do Peixe National Park in southern Brazil, state of Rio Grande do Sul, and they were found to be positive for CoVs. Phylogenetic analysis has demonstrated that these positives belong to two genera of the CoV. Gamma‐CoV‐positive samples were collected from one individual *Calidris alba* and one individual *Calidris fuscicollis* belonging to a restricted species of sandpiper. Delta‐CoV‐positive samples were obtained from a black skimmer (*Rynchops niger*), carrying a strain that clustered similarly to the clade of viruses found in Ibirapuera Forest in a phylogenetic study (Barbosa et al., 2019). Although it is difficult to foresee, many migratory species may have past interaction with migratory birds from Western/Central Siberia, the Balkans, the Black Sea and Central Asia using East African flyways to enter wintering areas to rest along with the river systems that cross the Arab Peninsula and the Nile (Lima et al., 2015). The direct transfer of an infectious agent is rarely reported from wild birds to humans. Potential causes and strategies for the spread of infectious agents from birds to humans require further exploration (Gilbert et al., 2006; Olsen et al., 2006; Reed et al., 2003; Tsiodras et al., 2008; Verhagen et al., 2015).

## 5 CONCLUSION

Globally, CoVs are widespread in several species of wild birds. The data on the prevalence of CoVs in wild birds across the world are scarce. Wild bird species of certain countries or locations are linked by one or more bird migration routes, which may encourage the spreading of native CoVs to the global wild bird and other animal populations. Moreover, interspecies transmission poses a great risk for spreading, mutation and the emergence of new strains of the viruses. To recognize their contribution to evolving novel viruses and zoonotic diseases, region‐specific and outcome‐based studies need to be conducted immediately with continuous surveillance of wild birds.

## CONFLICT OF INTEREST

The authors announce that there is no conflict of interest.

## AUTHOR CONTRIBUTION

**Mijanur Rahman, Md.:** Conceptualization; Data curation; Formal analysis; Methodology; Project administration; Supervision; Writing‐original draft; Writing‐review & editing. **Asma Talukder:** Conceptualization; Data curation; Formal analysis; Methodology; Project administration; Writing‐original draft; Writing‐review & editing. **Mohammed Mehadi Hassan Chowdhury:** Conceptualization; Data curation; Formal analysis; Writing‐review & editing. **Reshma Talukder:** Data curation; Graphical Illustration; Software; Writing‐original draft. **Rekha Akter:** Data curation; Formal analysis; Investigation; Methodology; Project administration; Writing‐original draft.

### PEER REVIEW

The peer review history for this article is available at https://publons.com/publon/10.1002/vms3.360.
